# Existence results for a coupled system of nonlinear fractional multi-point boundary value problems at resonance

**DOI:** 10.1186/s13660-018-1792-x

**Published:** 2018-07-28

**Authors:** Yinghan Zhang

**Affiliations:** College of Mathematics and Systems Science, Shandong University of Science and Technology, Qingdao, P.R. China

**Keywords:** 34A08, 34B10, Coupled system, Fractional differential equations, At resonance, Coincidence degree

## Abstract

By using the coincidence degree theory, we present an existence result for a coupled system of nonlinear fractional differential equations with multi-point boundary conditions at resonance.

## Introduction

In recent years, due to the application in many fields, fractional calculus has attracted more and more attention from researchers, and many meaningful results have been obtained [1, 2, 9–14, 17, 18, 24–26]. Since some nature phenomena are naturally modeled by fractional differential equation boundary value problems, it is important to study the problems of nonlinear fractional differential equations boundary value problems. On the other hand, the study of a coupled system involving fractional differential equations boundary value problems is also important as such systems occur in various problems of applied nature, for instance, see [2, 22, 31].

Recently, Su [31] discussed a two-point boundary value problem for a coupled system of fractional differential equations

$$ \textstyle\begin{cases} D_{0+}^{\alpha}u(t) =f(t,\upsilon(t),D_{0+}^{\mu}\upsilon(t)), \quad 0< t< 1, \\ D_{0+}^{\beta}\upsilon(t) =g(t,u(t),D_{0+}^{\nu}u(t)), \quad 0< t< 1,\\ u(0)=u(1)=\upsilon(0)=\upsilon(1)=0, \end{cases} $$

where $1<\alpha,\beta<2,\mu,\nu>0,\alpha-\nu\geq1,\beta-\mu \geq1, f,g:[0,1]\times\mathbb{R}\times\mathbb{R}\rightarrow\mathbb{R}$ are given functions and $D_{0+}^{\alpha}$ is the standard Riemann–Liouville derivative of order *α*. Bashir Ahmad et al. [2] considered a three-point boundary value problem for a coupled system of nonlinear fractional differential equations given by

$$ \textstyle\begin{cases} D_{0+}^{\alpha}u(t) =f(t,\upsilon(t),D_{0+}^{p} \upsilon(t)), \quad 0< t< 1, \\ D_{0+}^{\beta}\upsilon(t) =g(t,u(t),D_{0+}^{q} u(t)), \quad 0< t< 1,\\ u(0)=\upsilon(0)=0,\qquad u(1)=\gamma u(\eta),\qquad \upsilon(1)=\gamma \upsilon(\eta), \end{cases} $$

where $1<\alpha,\beta<2,p,q,\gamma>0,0<\eta<1,\alpha-q\geq1,\beta -p\geq1, \gamma\eta^{\alpha-1}<1,\gamma\eta^{\beta-1}<1, f,g:[0,1]\times\mathbb{R}\times\mathbb{R}\rightarrow\mathbb{R}$ are given functions and $D_{0+}^{\alpha}$ is the standard Riemann–Liouville derivative of order *α*. For more fractional-order boundary value problems and boundary value problems, we refer the reader to [3, 4, 6, 8, 15, 16, 19–21, 23, 27, 29, 30, 32, 33, 36].

It should be noted that all of the above papers mentioned deal with non-resonance case. However, there are few papers that consider the coupled system of nonlinear fractional differential equations with boundary conditions at resonance. In [5], Bai investigated the nonlinear nonlocal problem

$$\begin{aligned} D_{0+}^{\alpha}u(t) =f \bigl(t,u(t) \bigr),\qquad u(0)=0,\qquad \beta u( \eta)=u(1),\quad 0< t< 1, \end{aligned}$$

where $1<\alpha\leq2,0<\beta\eta^{\alpha-1}<1$. If $\beta\eta ^{\alpha-1}=1$, resonance occurs, this case was considered in [34]. In [34] the authors considered the existence of solutions of the fractional order ordinary differential equation

$$\begin{aligned} D_{0+}^{\alpha}u(t) =f \bigl(t,u(t),D_{0+}^{\alpha-1}u(t) \bigr)+e(t),\quad 0< t< 1, \end{aligned}$$

with boundary value conditions $I_{0+}^{2-\alpha}u(0)=0, u(1)=\sigma u(\eta)$, where $1< \alpha\leq2$ is a real number, $D_{0+}^{\alpha}$ and $I_{0+}^{\alpha}$ are the standard Riemann–Liouville derivative and integral, respectively, and $\sigma\eta^{\alpha-1}=1$. Under such conditions, the kernel of the linear operator $L=D_{0+}^{\alpha}$ is of one dimension. The case that the kernel of the linear operator $L=D_{0+}^{\alpha}$ is of two dimensions was considered in [7].

In [22], Jiang studied the solvability for a coupled system of fractional differential equations at resonance. In [35], the authors investigated a three-point boundary value problem for a coupled system of nonlinear fractional differential equations given by

1.1 $$ \textstyle\begin{cases} D_{0+}^{\alpha}u(t) =f(t,\upsilon(t),D_{0+}^{\beta-1}\upsilon(t)),\quad 0< t< 1,\\ D_{0+}^{\beta}\upsilon(t) =g(t,u(t),D_{0+}^{\alpha-1}u(t)),\quad 0< t< 1,\\ u(0)=\upsilon(0)=0,\qquad u(1)=\sigma_{1}u(\eta_{1}),\qquad \upsilon(1)=\sigma _{2}\upsilon(\eta_{2}), \end{cases} $$

where $1< \alpha,\beta\leq 2,0<\eta_{1},\eta_{2}<1,\sigma_{1},\sigma_{2}>0,\sigma_{1}\eta_{1}^{\alpha -1}=\sigma_{2}\eta_{2}^{\beta-1}=1$, *D* is the standard Riemann–Liouville fractional derivative. System (1.1) happens to be at resonance in the sense that the associated linear homogeneous coupled system

$$ \textstyle\begin{cases} D_{0+}^{\alpha}u(t) =0,\qquad D_{0+}^{\beta}\upsilon(t) =0,\quad 0< t< 1,\\ u(0)=\upsilon(0)=0,\qquad u(1)=\sigma_{1}u(\eta_{1}),\qquad \upsilon(1)=\sigma _{2}\upsilon(\eta_{2}), \end{cases} $$

has $(u(t),\upsilon(t))=(c_{1}t^{\alpha-1},c_{2}t^{\beta-1}), c_{1},c_{2}\in\mathbb{R}$ as a nontrivial solution.

Enlightened by the above contributions, in this paper we investigate the multi-point boundary value problem at resonance for a coupled system of nonlinear fractional differential equations given by

1.2 $$\begin{aligned} &\textstyle\begin{cases} D_{0+}^{\alpha}u(t) =f(t,\upsilon(t),D_{0+}^{\beta-2}\upsilon (t),D_{0+}^{\beta-1}\upsilon(t)),& 0< t< 1,\\ D_{0+}^{\beta}\upsilon(t) =g(t,u(t),D_{0+}^{\alpha -2}u(t),D_{0+}^{\alpha-1}u(t)),&0< t< 1, \end{cases}\displaystyle \end{aligned}$$

1.3 $$\begin{aligned} & \textstyle\begin{cases} u(0)=0, \qquad D_{0+}^{\alpha-1}u(0)=D_{0+}^{\alpha-1}u(\eta),\qquad u(1)=\sum_{i=1}^{m_{1}}\alpha_{i}u(\eta_{i}),\\ \upsilon(0)=0,\qquad D_{0+}^{\beta-1}\upsilon(0)=D_{0+}^{\beta-1}u(\xi),\qquad \upsilon(1)=\sum_{i=1}^{m_{2}}\beta_{i}\upsilon(\xi_{i}), \end{cases}\displaystyle \end{aligned}$$

where $2< \alpha,\beta\leq3,0<\xi,\eta\leq1, 0<\eta_{i},\xi _{j}<1\ (1\leq i\leq m_{1},1\leq j\leq m_{2}),m_{1}\geq2,m_{2}\geq2$, and $f,g:[0,1]\times\mathbb{R}^{3}\rightarrow\mathbb{R}$ satisfy the Carathéodory conditions. $D_{0+}^{\alpha}$ and $I_{0+}^{\alpha}$ are the standard Riemann–Liouville fractional derivative and fractional integral, respectively, and

1.4 $$\begin{aligned} &\sum_{i=1}^{m_{1}} \alpha_{i} \eta_{i}^{\alpha-1}=\sum_{i=1}^{m_{1}} \alpha_{i}\eta_{i}^{\alpha-2}=1, \end{aligned}$$

1.5 $$\begin{aligned} &\sum_{i=1}^{m_{2}} \beta_{i} \xi_{i}^{\beta-1}=\sum_{i=1}^{m_{2}} \beta_{i}\xi_{i}^{\beta-2}=1. \end{aligned}$$

We assume, in addition, that

1.6 $$\begin{aligned} R^{\alpha}={}&\frac{1}{\alpha}\eta^{\alpha}\frac{\Gamma(\alpha )\Gamma(\alpha-1)}{\Gamma(2\alpha-1)} \Biggl[1-\sum_{i=1}^{m_{1}} \alpha_{i}\eta_{i}^{2\alpha-2} \Biggr] \\ &{}-{}\frac{1}{\alpha-1}\eta^{\alpha-1}\frac{(\Gamma(\alpha ))^{2}}{\Gamma(2\alpha)} \Biggl[1-\sum _{i=1}^{m_{1}} \alpha_{i} \eta_{i}^{2\alpha-1} \Biggr] \neq0, \end{aligned}$$

1.7 $$\begin{aligned} R^{\beta}={}&\frac{1}{\beta}\xi^{\beta}\frac{\Gamma(\beta)\Gamma (\beta-1)}{\Gamma(2\beta-1)} \Biggl[1-\sum_{i=1}^{m_{2}} \alpha_{i}\eta_{i}^{2\alpha-2} \Biggr] \\ &{}-\frac{1}{\beta-1}\xi^{\beta-1}\frac{(\Gamma(\beta ))^{2}}{\Gamma(2\beta)} \Biggl[1-\sum _{i=1}^{m_{2}} \beta_{i} \xi_{i}^{2\beta-1} \Biggr] \neq0, \end{aligned}$$

where Γ is the gamma function. Due to conditions (1.4) and (1.5), the coupled system (1.2)–(1.3) happens to be at resonance in the sense that the associated linear homogeneous coupled system

$$ \textstyle\begin{cases} D_{0+}^{\alpha}u(t) =0,&0< t< 1,\\ D_{0+}^{\beta}\upsilon(t) =0,&0< t< 1, \end{cases} $$

with boundary value conditions (1.3) has $(u(t),\upsilon(t))=(c_{11}t^{\alpha-1}+c_{12}t^{\alpha -2},c_{21}t^{\beta-1}+ c_{22}t^{\beta-2}), c_{ij}\in\mathbb{R}$ as nontrivial solutions.

Since the associated linear homogeneous coupled system about (1.2)–(1.3) has nontrivial solutions $(u(t),\upsilon (t))=(c_{11}t^{\alpha-1}+c_{12}t^{\alpha-2},c_{21}t^{\beta -1}+c_{22}t^{\beta-2}), c_{ij}\in\mathbb{R}$, it is more complex than [35].

The rest of this paper is organized as follows. We present some notations and lemmas in Sect. 2 and establish a theorem of existence of a solution for the coupled system (1.2)–(1.3) in Sect. 3.

## Background materials and methods

In this section, we present some necessary basic knowledge about fractional calculus theory and a fixed point theorem.

The definitions and properties of fractional integral and derivative can be found in many literature works [24].

### Definition 2.1

([24])

The Riemann–Liouville fractional integral $I_{0+}^{\alpha}f$ and derivative $D_{0+}^{\alpha}y$ of order $\alpha\ (\alpha>0)$ are defined by

$$ I_{0+}^{\alpha}f(t) = \frac{1}{\Gamma(\alpha)} \int_{0}^{t} (t-s)^{\alpha-1}f(s)\,\mathrm{d}s \quad (t>0), $$

and

$$ D_{0+}^{\alpha}y(t) = \frac{1}{\Gamma(n-\alpha)} \biggl( \frac{d}{dt} \biggr)^{n} \int_{0}^{t} \frac{y(s)}{(t-s)^{\alpha-n+1}}\,\mathrm{d}s, $$

respectively, where $n=[\alpha]+1$.

The properties of fractional calculus we will use are listed below.

Assume that $u\in C(0,1) \cap L^{1}(0,1)$ and $D_{0+}^{\alpha}u\in C(0,1) \cap L^{1}(0,1)$ with $\alpha> 0$. Then

$$ I_{0+}^{\alpha}D_{0+}^{\alpha}u(t) = u(t)+C_{1}t^{\alpha-1}+C_{2}t^{\alpha-2}+ \cdots+C_{N}t^{\alpha-N}, $$

where $C_{i}\in\mathbb{R},i=1,2,\ldots,N$, $N=[\alpha+1]-1$. If $\alpha>0,\beta>0$, then for a continuous function *f*, the equality $(I_{0+}^{\alpha}I_{0+}^{\beta}f)(x)=(I_{0+}^{\alpha+\beta}f)(x)$ is satisfied. Let $\alpha>0,m\in\mathbb{N}$, and $D=d/dx$. If the fractional derivatives $(D_{0+}^{\alpha}y)(x)$ and $(D_{0+}^{\alpha+m}y)(x)$ exist, then $(D^{m}D_{0+}^{\alpha}y)(x)=(D_{0+}^{\alpha+m}y)(x)$. If $\alpha>0$, then for a continuous function *f*, $(D_{0+}^{\alpha}I_{0+}^{\alpha}f)(x)=f(x)$ is satisfied. If $\alpha>\beta>0$, then for a continuous function *f*, it has $(D_{0+}^{\beta}I_{0+}^{\alpha}f)(x)=(I_{0+}^{\alpha-\beta}f)(x)$.

Now, we present some notations and a fixed point theorem.

Let $Y,Z$ be real Banach spaces, $L:\operatorname{dom}(L)\subset Y\rightarrow Z$ be a Fredholm map of index zero, and $P:Y\rightarrow Y,Q:Z\rightarrow Z$ be continuous projectors such that

$$\begin{aligned} &\operatorname{Im}(P)=\operatorname{Ker}(L),\qquad \operatorname{Ker}(Q)= \operatorname{Im}(L), \\ &Y=\operatorname{Ker}(L)\oplus\operatorname{Ker}(P),\qquad Z= \operatorname{Im}(L)\oplus \operatorname{Im}(Q). \end{aligned}$$

We can conclude that $L|_{\operatorname{dom}(L)\cap\operatorname{Ker}(P)}:\operatorname {dom}(L)\cap\operatorname{Ker}(P)\rightarrow \operatorname{Im}(L)$ is invertible. Denote the inverse of the map by $K_{P}$. If Ω is an open bounded subset of *Y* such that $\operatorname{dom}(L)\cap \Omega\neq \emptyset$ and $QN(\overline{\Omega})$ is bounded and $K_{P}(I-Q)N:\overline{\Omega}\rightarrow Y$ is compact, then the map $N:Y\rightarrow Z$ will be called *L*-compact on Ω̅.

The theorem we used is Theorem 2.4 of [28].

### Theorem 2.1

*Suppose that* *L* *is a Fredholm operator of index zero and* *N* *is L*-*compact on* Ω̅, *and the following conditions are satisfied*:

(i) $Lx \neq\lambda Nx$ *for every* $(x,\lambda) \in [(\operatorname{dom}(L)\backslash\operatorname{Ker}(L))\cap\partial \Omega]\times(0,1)$;
(ii) $Nx\notin \operatorname{Im}(L)$ *for every* $x\in\operatorname {Ker}(L)\cap\partial\Omega$;
(iii) $\operatorname{deg} (JQN|_{\operatorname {Ker}(L)},\Omega\cap\operatorname{Ker}(L),0 )\neq0$, *where* $Q:Z\rightarrow Z$ *is a projection as above with* $\operatorname{Im}(L)=\operatorname{Ker}(Q)$ *and* $J:\operatorname{Im}(Q)\rightarrow\operatorname {Ker}(L)$ *is any isomorphism*.

*Then the equation* $Lx=Nx$ *has at least one solution in* $\operatorname {dom}(L)\cap\overline{\Omega}$.

In the following, the Banach space $C[0,1]$ with the norm $\|x\|_{\infty}=\max_{t\in[0,1]}|x(t)|$ and $L^{1}[0,1]$ with the norm $\|x\|_{1}=\int_{0}^{1}|x(t)|\,\mathrm{d}t$ will be used. Given $\mu>0$ and $N=[\mu]+1$, one can define a linear space

2.1 $$ C^{\mu}[0,1]:= \bigl\{ u(t)|u(t)=I_{0+}^{\mu}x(t)+c_{1}t^{\mu-1}+\cdots+ c_{N-1}t^{\mu-(N-1)},t \in[0,1] \bigr\} , $$

where $x\in C[0,1]$ and $c_{i}\in \mathbb{R},i=1,\ldots,N-1$. It can prove that with the norm $\|u\|_{C^{\mu}}=\|D_{0+}^{\mu}u\|_{\infty}+\cdots+\|D_{0+}^{\mu-(N-1)} u\|_{\infty}+ \|u\|_{\infty}$, $C^{\mu}[0,1]$ is a Banach space [34].

### Lemma 2.1

([34])

$F\subset C^{\mu}[0,1]$ *is a sequentially compact set if and only if* *F* *is uniformly bounded and equicontinuous*. *Here uniformly bounded means that there exists* $M>0$ *such that*, *for every* $u\in F$, *it has*

$$\Vert u \Vert _{C^{\mu}}= \bigl\Vert D_{0+}^{\mu}u \bigr\Vert _{\infty}+\cdots+ \bigl\Vert D_{0+}^{\mu-(N-1)} u \bigr\Vert _{\infty}+ \Vert u \Vert _{\infty}< M, $$

*and equicontinuous means that for* $\forall\varepsilon>0$, $\exists\delta>0$ *such that*, *for any* $t_{1},t_{2}\in[0,1]$,

$$\begin{aligned} & \bigl\vert u(t_{1})-u(t_{2}) \bigr\vert < \varepsilon\quad \bigl(\forall u \in F, \vert t_{1}-t_{2} \vert< \delta \bigr), \\ & \bigl\vert D_{0+}^{\alpha-i}u(t_{1})-D_{0+}^{\alpha-i}u(t_{2}) \bigr\vert < \varepsilon\quad \bigl(\forall u\in F,\forall i\in\{0,\ldots ,N-1\}, \vert t_{1}-t_{2} \vert< \delta \bigr). \end{aligned}$$

Let $Z_{1}=L^{1}[0,1]$ with the norm $\|\cdot\|_{1}$. Fractional functional spaces $Y_{1}=C^{\alpha-1}[0,1]$ and $Y_{2}=C^{\beta-1}[0,1]$ defined by (2.1) are equipped with the norms $\|u\|_{Y_{1}}=\|D_{0+}^{\alpha-1} u\|_{\infty}+\|D_{0+}^{\alpha-2} u\| _{\infty}+\|u\|_{\infty}$ and $\|\upsilon\|_{Y_{2}}=\|D_{0+}^{\beta-1}\upsilon\|_{\infty}+\| D_{0+}^{\beta-2}\upsilon\|_{\infty}+\|\upsilon\|_{\infty}$, respectively. Then $Y=Y_{1}\times Y_{2}$ is a Banach space with the norm defined by $\|(u,\upsilon)\|_{Y}=\max\{\|u\|_{Y_{1}},\|\upsilon\|_{Y_{2}}\}$ and $Z=Z_{1}\times Z_{1}$ is a Banach space with the norm defined by $\|(x,y)\|_{Z}=\max\{\|x\|_{1},\|y\|_{1}\}$.

### Definition 2.2

A pair of functions $(u,\upsilon)\in Y$ is called a solution of the coupled system of multi-point boundary value problem (1.2)–(1.3) if $D_{0+}^{\alpha-1}u$ and $D_{0+}^{\beta -1}\upsilon$ are all absolutely continuous on $(0,1)$, $(u,\upsilon)$ satisfies (1.2) almost everywhere on $(0,1)$ and satisfies boundary conditions (1.3).

### Definition 2.3

The map $f:[0,1]\times\mathbb{R}^{n}\rightarrow \mathbb{R}$ satisfies the Carathéodory conditions with respect to $L^{1}[0,1]$ if the following conditions hold:

(i) for each $z\in\mathbb{R}^{n}$, the mapping $t\rightarrow f(t,z)$ is Lebesgue measurable;
(ii) for almost each $t\in[0,1]$, the mapping $z\rightarrow f(t,z)$ is continuous on $\mathbb{R}^{n}$;
(iii) for each $r>0$, there exists $\rho_{r}\in L^{1}([0,1],\mathbb{R})$ such that, for a.e. $t\in[0,1]$ and every $|z|\leq r$, we have $|f(t,z)|\leq\rho_{r}(t)$.

Define $L_{1}: \operatorname{dom}(L_{1})\cap Y_{1}\rightarrow Z_{1}$ by

2.2 $$ L_{1}u=D_{0+}^{\alpha}u,\quad u\in \operatorname{dom}(L_{1}), $$

with

$$\begin{aligned} \operatorname{dom}(L_{1})={}& \Biggl\{ u\in C^{\alpha -1}[0,1]|D_{0+}^{\alpha}u\in L^{1}[0,1],u(0)=0, \\ &{}D_{0+}^{\alpha-1}u(0)=D_{0+}^{\alpha-1}u( \eta),u(1)=\sum_{i=1}^{m_{1}} \alpha_{i}u(\eta_{i}) \Biggr\} . \end{aligned}$$

Define $L_{2}: \operatorname{dom}(L_{2})\cap Y_{2}\rightarrow Z_{1}$ by

2.3 $$ L_{2}\upsilon=D_{0+}^{\beta}\upsilon,\quad \upsilon \in \operatorname{dom}(L_{2}), $$

with

$$\begin{aligned} \operatorname{dom}(L_{2})={}& \Biggl\{ \upsilon\in C^{\beta -1}[0,1]|D_{0+}^{\beta}\upsilon\in L^{1}[0,1],\upsilon(0)=0, \\ &{}D_{0+}^{\beta-1}\upsilon(0)=D_{0+}^{\beta-1} \upsilon(\xi),\upsilon(1)=\sum_{i=1}^{m_{2}} \beta_{i}\upsilon(\xi_{i}) \Biggr\} . \end{aligned}$$

Define *L* to be the linear operator from $\operatorname{dom}(L)\cap Y$ to *Z* with

$$\operatorname{dom}(L)= \bigl\{ (u,\upsilon)\in Y|u\in\operatorname {dom}(L_{1}),\upsilon\in\operatorname{dom}(L_{2}) \bigr\} , $$

and

2.4 $$ L(u,\upsilon)=(L_{1} u,L_{2} \upsilon),\quad (u,\upsilon)\in \operatorname{dom}(L). $$

Define $N:Y\rightarrow Z$ as

2.5 $$ N(u,\upsilon)=(N_{1}\upsilon,N_{2}u), $$

where $N_{1}:Y_{2}\rightarrow Z_{1}$ is defined by

2.6 $$ N_{1}\upsilon(t)=f \bigl(t,\upsilon(t),D_{0+}^{\beta-2} \upsilon(t),D_{0+}^{\beta-1}\upsilon(t) \bigr), $$

and $N_{2}:Y_{1}\rightarrow Z_{1}$ is defined by

2.7 $$ N_{2}u(t)=g \bigl(t,u(t),D_{0+}^{\alpha -2}u(t),D_{0+}^{\alpha-1}u(t) \bigr). $$

Then the coupled system of boundary value problem (1.2)–(1.3) can be written by

2.8 $$\begin{aligned} L(u,\upsilon)=N(u,\upsilon). \end{aligned}$$

### Lemma 2.2

*The mapping* $L:\operatorname{dom}(L)\subset Y\rightarrow Z$ *is a Fredholm operator of index zero*.

### Proof

It is clear that $\operatorname{Ker}(L)=\{(c_{11}t^{\alpha-1}+c_{12}t^{\alpha -2},c_{21}t^{\beta-1}+c_{22}t^{\beta-2})|c_{ij}\in \mathbb{R},i,j=1,2\}\cong\mathbb{R}^{4}$.

Let $(x,y)\in \operatorname{Im}(L)$, then there exists $(u,\upsilon)\in\operatorname {dom}(L)$ such that $(x,y)=L(u,\upsilon)$, that is, $u\in Y_{1},x=D_{0+}^{\alpha}u$, and $\upsilon\in Y_{2},y=D_{0+}^{\beta}\upsilon$. Thus we have

$$ I_{0+}^{\alpha}x(t)=u(t) + c_{1}t^{\alpha-1}+c_{2}t^{\alpha -2}+c_{3}t^{\alpha-3}. $$

By the boundary conditions (1.3), we obtain $c_{3}=0$, $c_{1},c_{2}$ can be any constants, and *x* satisfies

$$\begin{aligned} D_{0+}^{\alpha-1}I_{0+}^{\alpha}x(0)=D_{0+}^{\alpha-1}I_{0+}^{\alpha}x( \eta), \qquad I_{0+}^{\alpha}x(1)=\sum_{i=1}^{m_{1}} \alpha_{i}I_{0+}^{\alpha}x(\eta_{i}). \end{aligned}$$

By the properties of fractional calculus, $D_{0+}^{\alpha -1}I_{0+}^{\alpha}=I_{0+}^{1}$, thus the above two equalities can be reduced to

2.9 $$\begin{aligned} & \int_{0}^{\eta}x(s)\,\mathrm{d}s=0, \end{aligned}$$

2.10 $$\begin{aligned} & \int_{0}^{1}(1-s)^{\alpha-1}x(s)\,\mathrm{d}s- \sum_{i=1}^{m_{1}}\alpha_{i} \int_{0}^{\eta_{i}}(\eta_{i}-s)^{\alpha-1}x(s) \,\mathrm{d}s=0. \end{aligned}$$

Similarly, we can derive that *y* satisfies

2.11 $$\begin{aligned} & \int_{0}^{\xi}y(s)\,\mathrm{d}s=0, \end{aligned}$$

2.12 $$\begin{aligned} & \int_{0}^{1}(1-s)^{\beta-1}y(s)\,\mathrm{d}s- \sum_{i=1}^{m_{2}}\beta_{i} \int_{0}^{\xi_{i}}(\xi_{i}-s)^{\beta-1}y(s) \,\mathrm{d}s=0. \end{aligned}$$

On the other hand, suppose that $x,y\in Z_{1}$ satisfy (2.9)–(2.10) and (2.11)–(2.12), respectively. Let $u(t)=I_{0+}^{\alpha}x(t),\upsilon(t)=I_{0+}^{\beta}y(t)$, then a basic calculation shows that $u\in\operatorname{dom}(L_{1})$, $D_{0+}^{\alpha}u(t) =x(t)$, and $\upsilon\in\operatorname {dom}(L_{2}),D_{0+}^{\beta}\upsilon(t) =y(t)$. That is, $(x,y)\in \operatorname{Im}(L)$. From the above argument, we can derive that

2.13 $$ \operatorname{Im}(L)= \bigl\{ (x,y)\in Z|x \mbox{ satisfies (2.9)--(2.10)},y \mbox{ satisfies (2.11)--(2.12)} \bigr\} . $$

Consider continuous linear mappings $Q_{1}^{\alpha}:Z_{1}\rightarrow Z_{1}$ and $Q_{2}^{\alpha}:Z_{1}\rightarrow Z_{1}$ defined by

2.14 $$\begin{aligned} &Q^{\alpha}_{1}x= \int_{0}^{\eta}x(s)\,\mathrm{d}s, \end{aligned}$$

2.15 $$\begin{aligned} &Q^{\alpha}_{2}x= \int_{0}^{1}(1-s)^{\alpha-1}x(s)\,\mathrm{d}s- \sum_{i=1}^{m_{1}}\alpha_{i} \int_{0}^{\eta_{i}}(\eta_{i}-s)^{\alpha-1}x(s) \,\mathrm{d}s. \end{aligned}$$

Continuous linear mappings $Q^{\beta}_{1}:Z_{1}\rightarrow Z_{1}$ and $Q_{2}^{\beta}:Z_{1}\rightarrow Z_{1}$ are defined by

2.16 $$\begin{aligned} &Q^{\beta}_{1}x= \int_{0}^{\xi}x(s)\,\mathrm{d}s, \end{aligned}$$

2.17 $$\begin{aligned} &Q^{\beta}_{2}x= \int_{0}^{1}(1-s)^{\beta-1}x(s)\,\mathrm{d}s- \sum_{i=1}^{m_{2}}\beta_{i} \int_{0}^{\xi_{i}}(\xi_{i}-s)^{\beta-1}x(s) \,\mathrm{d}s. \end{aligned}$$

Using the above definitions, we construct the following auxiliary maps $R^{\alpha}_{1},R^{\alpha}_{2},R^{\beta}_{1}$, and $R^{\beta}_{2}:Z_{1}\rightarrow Z_{1}$ by

2.18 $$\begin{aligned} &R_{1}^{\alpha}g=\frac{1}{R^{\alpha}} \Biggl[\frac{\Gamma(\alpha )\Gamma(\alpha-1)}{\Gamma(2\alpha-1)} \Biggl(1-\sum_{i=1}^{m_{1}} \alpha_{i}\eta_{i}^{2\alpha-2} \Biggr)Q^{\alpha}_{1}g- \frac{1}{\alpha-1}\eta^{\alpha-1}Q^{\alpha}_{2}g \Biggr], \end{aligned}$$

2.19 $$\begin{aligned} &R^{\alpha}_{2}g=-\frac{1}{R^{\alpha}} \Biggl[\frac{(\Gamma(\alpha ))^{2}}{\Gamma(2\alpha)} \Biggl(1-\sum_{i=1}^{m_{1}} \alpha_{i}\eta_{i}^{2\alpha-1} \Biggr) Q_{1}^{\alpha}g-\frac{1}{\alpha}\eta^{\alpha}Q^{\alpha}_{2}g \Biggr], \end{aligned}$$

2.20 $$\begin{aligned} &R_{1}^{\beta}g=\frac{1}{R^{\beta}} \Biggl[\frac{\Gamma(\beta)\Gamma (\beta-1)}{\Gamma(2\beta-1)} \Biggl(1-\sum_{i=1}^{m_{2}} \beta_{i} \xi_{i}^{2\beta-2} \Biggr)Q^{\beta}_{1}g- \frac{1}{\beta-1}\xi^{\beta-1}Q^{\beta}_{2}g \Biggr], \end{aligned}$$

2.21 $$\begin{aligned} &R_{2}^{\beta}g=-\frac{1}{R^{\beta}} \Biggl[\frac{(\Gamma(\beta ))^{2}}{\Gamma(2\beta)} \Biggl(1-\sum_{i=1}^{m_{2}} \beta_{i} \xi_{i}^{2\beta-1} \Biggr) Q_{1}^{\beta}g- \frac{1}{\beta}\xi^{\beta}Q^{\alpha}_{2}g \Biggr]. \end{aligned}$$

Since conditions (1.6)–(1.7) hold, the mappings $Q^{\alpha}:Z_{1}\rightarrow Z_{1}$ and $Q^{\beta}:Z_{1}\rightarrow Z_{1}$ defined by

2.22 $$\begin{aligned} &Q^{\alpha}g= \bigl(R_{1}^{\alpha}g \bigr)t^{\alpha-1}+ \bigl(R_{2}^{\alpha}g \bigr)t^{\alpha-2}, \end{aligned}$$

2.23 $$\begin{aligned} &Q^{\beta}g= \bigl(R_{1}^{\beta}g \bigr)t^{\beta-1}+ \bigl(R_{2}^{\beta}g \bigr)t^{\beta-2} \end{aligned}$$

are well defined. Thus, we can define the continuous linear mapping $Q:Z\rightarrow Z$ by

2.24 $$ Q(x,y)= \bigl(Q^{\alpha}x,Q^{\beta}y \bigr). $$

Recall (1.6) and note that

2.25 $$\begin{aligned} & R_{1}^{\alpha}\bigl(R_{1}^{\alpha}gt^{\alpha-1} \bigr) \\ &\quad=\frac{1}{R^{\alpha}} \Biggl[\frac{\Gamma(\alpha)\Gamma(\alpha -1)}{\Gamma(2\alpha-1)} \Biggl(1-\sum _{i=1}^{m_{1}} \alpha_{i}\eta_{i}^{2\alpha-2} \Biggr)Q_{1}^{\alpha}\bigl(R^{\alpha}_{1}gt^{\alpha-1} \bigr) -\frac{1}{\alpha-1}\eta^{\alpha-1}Q^{\alpha}_{2} \bigl(R^{\alpha}_{1}gt^{\alpha-1} \bigr) \Biggr] \\ &\quad=R^{\alpha}_{1}g\frac{1}{R^{\alpha}} \Biggl[ \frac{\Gamma(\alpha )\Gamma(\alpha-1)}{\Gamma(2\alpha-1)} \Biggl(1-\sum_{i=1}^{m_{1}} \alpha_{i}\eta_{i}^{2\alpha-2} \Biggr)\frac{1}{\alpha} \eta^{\alpha} \\ &\qquad{}-\frac{1}{\alpha-1}\eta^{\alpha-1}\frac{(\Gamma(\alpha ))^{2}}{\Gamma(2\alpha)} \Biggl(1- \sum _{i=1}^{m_{1}} \alpha_{i} \eta_{i}^{2\alpha-1} \Biggr) \Biggr]=R^{\alpha}_{1}g. \end{aligned}$$

Similarly, we can derive that $R^{\alpha}_{1}(R^{\alpha}_{2}gt^{\alpha -2})=0$ and

$$\begin{aligned} &R^{\alpha}_{2} \bigl(R^{\alpha}_{1}gt^{\alpha-1} \bigr)=0,\qquad R^{\alpha}_{2} \bigl(R^{\alpha}_{2}gt^{\alpha-2} \bigr)=R^{\alpha}_{2}g, \qquad R^{\beta}_{1} \bigl(R^{\beta}_{1}gt^{\alpha-1} \bigr)=R^{\beta}_{1}g, \\ &R^{\beta}_{1} \bigl(R^{\beta}_{2}gt^{\beta-2} \bigr)=0,\qquad R^{\beta}_{2} \bigl(R^{\beta}_{1}gt^{\beta-1} \bigr)=0,\qquad R^{\beta}_{2} \bigl(R^{\beta}_{2}gt^{\beta-2} \bigr)=R^{\beta}_{2}g. \end{aligned}$$

For $(x,y)\in Z$, it follows from the above relations that

2.26 $$\begin{aligned} Q^{2}(x,y)&= \bigl(Q^{\alpha}Q^{\alpha}x,Q^{\beta}Q^{\beta}y \bigr) \\ &= \bigl(Q^{\alpha}\bigl( \bigl(R_{1}^{\alpha}x \bigr)t^{\alpha-1}+ \bigl(R_{2}^{\alpha}x \bigr)t^{\alpha-2} \bigr),Q^{\beta}\bigl( \bigl(R_{1}^{\beta}y \bigr)t^{\beta-1}+ \bigl(R_{2}^{\beta}y \bigr)t^{\beta-2} \bigr) \bigr) \\ &= \bigl( \bigl(R^{\alpha}_{1}x \bigr)t^{\alpha-1}+ \bigl(R^{\alpha}_{2}x \bigr)t^{\alpha-2}, \bigl(R^{\beta}_{1}y \bigr)t^{\beta-1}+ \bigl(R^{\beta}_{2}y \bigr)t^{\beta-2} \bigr) \\ &= \bigl(Q^{\alpha}x,Q^{\beta}y \bigr)=Q(x,y), \end{aligned}$$

that is, the map *Q* is idempotent. In fact, *Q* is a continuous linear projector and $(x,y)\in \operatorname{Im}(L)$ is equivalent to $Q(x,y)=(0,0)$. In fact, $\operatorname{Im}(L)=\operatorname{Ker}(Q)$.

Take $(x,y)\in Z$ in the form $(x,y)=((x,y)-Q(x,y))+Q(x,y)$ so that $(x,y)-Q(x,y)\in \operatorname{Im}(L)=\operatorname{Ker}(Q)$ and $Q(x,y)\in \operatorname{Im}(Q)$. Thus, $Z=\operatorname{Im}(L)+\operatorname{Im}(Q)$. For every $(x,y)\in \operatorname{Im}(Q)$ has the form $(x,y)=(c_{11}t^{\alpha-1}+c_{12}t^{\alpha-2},c_{21}t^{\beta -1}+c_{22}t^{\beta-2}),c_{ij}\in\mathbb{R}\ (i,j=1,2)$. If $(x,y)=(c_{11}t^{\alpha-1}+c_{12}t^{\alpha-2},c_{21}t^{\beta -1}+c_{22}t^{\beta-2})$ satisfies (2.9)–(2.10) and (2.11)–(2.12) respectively, then we have $c_{ij}=0\ (i,j=1,2)$. Hence, $\operatorname{Im}(L)\cap \operatorname{Im}(Q)=(0,0)$ and, in fact, $Z=\operatorname{Im}(L)\oplus \operatorname{Im}(Q)$.

Now, $\operatorname{Ind}L= \operatorname{dim} \operatorname{Ker}(L)- \operatorname{codim} \operatorname{Im}(L)=0$, and so *L* is a Fredholm operator of index zero. □

Let the operators $P_{1}:Y_{1}\rightarrow Y_{1}$, $P_{2}:Y_{2}\rightarrow Y_{2}$, and $P:Y\rightarrow Y$ be defined by

2.27 $$\begin{aligned} & P_{1}u(t)=\frac{1}{\Gamma(\alpha)}D_{0+}^{\alpha -1}u(0)t^{\alpha-1}+ \frac{1}{\Gamma(\alpha-1)}D_{0+}^{\alpha-2}u(0)t^{\alpha-2}, \end{aligned}$$

2.28 $$\begin{aligned} &P_{2}\upsilon(t)=\frac{1}{\Gamma(\beta)}D_{0+}^{\beta-1} \upsilon(0)t^{\beta-1}+\frac{1}{\Gamma(\beta-1)}D_{0+}^{\beta-2} \upsilon(0)t^{\beta-2} \end{aligned}$$

and

2.29 $$ P(u,\upsilon)=(P_{1}u,P_{2}\upsilon), $$

respectively. Note that $P_{1},P_{2}$, and *P* are continuous linear projectors and

2.30 $$ \operatorname{Ker}(P)= \bigl(\operatorname {Ker}(P_{1}), \operatorname{Ker}(P_{2}) \bigr)= \bigl\{ (u,\upsilon)\in Y|D_{0+}^{\alpha-i}u(0)=D_{0+}^{\beta-i} \upsilon(0)=0,i=1,2 \bigr\} . $$

It is clear that $Y=\operatorname{Ker}(L)\oplus\operatorname {Ker}(P)$ and, for every $(u,\upsilon)\in Y$,

2.31 $$\begin{aligned} \bigl\Vert P(u,\upsilon) \bigr\Vert _{Y}={}& \bigl\Vert (P_{1}u,P_{2}\upsilon) \bigr\Vert _{Y}= \max \bigl\{ \Vert P_{1}u \Vert _{Y_{1}}, \Vert P_{2}\upsilon \Vert _{Y_{2}} \bigr\} \\ ={}&\max \biggl\{ \biggl\Vert \frac{1}{\Gamma(\alpha)} \bigl\vert D_{0+}^{\alpha-1}u(0) \bigr\vert t^{\alpha-1} + \frac{1}{\Gamma(\alpha-1)} \bigl\vert D_{0+}^{\alpha-2}u(0) \bigr\vert t^{\alpha-2} \biggr\Vert _{Y_{1}}, \\ & \biggl\Vert \frac{1}{\Gamma(\beta)} \bigl\vert D_{0+}^{\beta-1} \upsilon(0) \bigr\vert t^{\beta-1} +\frac{1}{\Gamma(\beta-1)} \bigl\vert D_{0+}^{\beta-2} \upsilon(0) \bigr\vert t^{\beta-2} \biggr\Vert _{Y_{2}} \biggr\} \\ \leq {}&\max \biggl\{ \biggl(2+\frac{1}{\Gamma(\alpha)} \biggr) \bigl\vert D_{0+}^{\alpha-1}u(0) \bigr\vert + \biggl(1+\frac{1}{\Gamma(\alpha-1)} \biggr) \bigl\vert D_{0+}^{\alpha-2}u(0) \bigr\vert , \\ &{} \biggl(2+\frac{1}{\Gamma(\beta)} \biggr) \bigl\vert D_{0+}^{\beta-1} \upsilon(0) \bigr\vert + \biggl(1+\frac{1}{\Gamma(\beta-1)} \biggr) \bigl\vert D_{0+}^{\beta-2} \upsilon(0) \bigr\vert \biggr\} . \end{aligned}$$

Define $K_{P}:\operatorname{Im}(L)\rightarrow\operatorname{dom}(L)\cap\operatorname {Ker}(P)$ by

2.32 $$ K_{P}(x,y)= \bigl(I_{0+}^{\alpha}x,I_{0+}^{\beta}y \bigr). $$

For $(x,y)\in \operatorname{Im}(L)$, we have

$$LK_{P}(x,y)=L\bigl(I_{0+}^{\alpha}x,I_{0+}^{\beta}y \bigr)= \bigl(D_{0+}^{\alpha}I_{0+}^{\alpha}x,D_{0+}^{\beta}I_{0+}^{\beta}y \bigr)=(x,y), $$

and for $(u,\upsilon)\in\operatorname{dom}(L)\cap\operatorname {Ker}(P)$, we have $u\in\operatorname{dom}(L_{1}),D_{0+}^{\alpha-1}u(0)=D_{0+}^{\alpha -2}u(0)=0$ and $\upsilon\in \operatorname{dom}(L_{2})$, $D_{0+}^{\beta-1}\upsilon(0)=D_{0+}^{\beta -2}\upsilon(0)=0$, so the coefficients $c_{1},c_{2},c_{3},c_{4},c_{5},c_{6}$ in the expressions $u=I_{0+}^{\alpha}D_{0+}^{\alpha}u+c_{1}t^{\alpha-1}+c_{2}t^{\alpha -2}+c_{3}t^{\alpha-3}$ and $\upsilon=I_{0+}^{\beta}D_{0+}^{\beta}\upsilon+c_{4}t^{\beta -1}+c_{5}t^{\beta-2}+c_{6}t^{\beta-3}$ are all equal to zero. Thus, we obtain

$$K_{P}L(u,\upsilon)=K_{P}(L_{1}u,L_{2} \upsilon)=\bigl(I_{0+}^{\alpha }D_{0+}^{\alpha}u,I_{0+}^{\beta}D_{0+}^{\beta} \upsilon\bigr) =(u,\upsilon). $$

This shows that $K_{P}=(L|_{\operatorname{dom}(L)\cap \operatorname{Ker}(P)})^{-1}$. Again, since for every $(x,y)\in \operatorname{Im}(L)$,

2.33 $$\begin{aligned} \bigl\Vert K_{P}(x,y) \bigr\Vert _{Y}={}& \bigl\Vert \bigl(I_{0+}^{\alpha}x,I_{0+}^{\beta}y \bigr) \bigr\Vert _{Y} =\max \bigl\{ \bigl\Vert I_{0+}^{\alpha}x \bigr\Vert _{Y_{1}}, \bigl\Vert I_{0+}^{\beta}y \bigr\Vert _{Y_{2}} \bigr\} \\ ={}&\max \bigl\{ \bigl\Vert I_{0+}^{\alpha}x \bigr\Vert _{\infty}+ \bigl\Vert D_{0+}^{\alpha-1}I_{0+}^{\alpha}x \bigr\Vert _{\infty}+ \bigl\Vert D_{0+}^{\alpha-2}I_{0+}^{\alpha}x \bigr\Vert _{\infty}, \\ &{} \bigl\Vert I_{0+}^{\beta}y \bigr\Vert _{\infty}+ \bigl\Vert D_{0+}^{\beta-1}I_{0+}^{\beta}y \bigr\Vert _{\infty}+ \bigl\Vert D_{0+}^{\beta-2}I_{0+}^{\beta}y \bigr\Vert _{\infty}\bigr\} \\ ={}&\max \bigl\{ \bigl\Vert I_{0+}^{\alpha}x \bigr\Vert _{\infty}+ \bigl\Vert I_{0+}^{1}x \bigr\Vert _{\infty}+ \bigl\Vert I_{0+}^{2}x \bigr\Vert _{\infty}, \bigl\Vert I_{0+}^{\beta}y \bigr\Vert _{\infty}+ \bigl\Vert I_{0+}^{1}y \bigr\Vert _{\infty}+ \bigl\Vert I_{0+}^{2}y \bigr\Vert _{\infty}\bigr\} \\ \leq{}&\max \biggl\{ \biggl(2+\frac{1}{\Gamma(\alpha)} \biggr) \Vert x \Vert _{1}, \biggl(2+\frac{1}{\Gamma(\beta)} \biggr) \Vert y \Vert _{1} \biggr\} . \end{aligned}$$

With arguments similar to those in [34], we obtain the following lemma.

### Lemma 2.3

$K_{P}(I-Q)N:Y\rightarrow Y$ *is completely continuous*.

## Results and discussion

In this section, we shall prove existence results for the coupled system of fractional boundary value problem (1.2)–(1.3).

For convenience, let us set the following notations:

3.1 $$ \textstyle\begin{cases} \rho_{1}=3+\frac{1}{\Gamma(\alpha)}+\frac{1}{\Gamma(\alpha-1)},\qquad \mu _{1}=2+\frac{1}{\Gamma(\alpha)},\qquad \epsilon_{1}=\rho_{1}+\mu_{1},\\ \rho_{2}=3+\frac{1}{\Gamma(\beta)}+\frac{1}{\Gamma(\beta-1)},\qquad \mu _{2}=2+\frac{1}{\Gamma(\beta)},\qquad \epsilon_{2}=\rho_{2}+\mu_{2}. \end{cases} $$

Assume that the following conditions are satisfied:

($H_{1}$)
There exists a constant $A>0$ such that, for $(u,\upsilon)\in\operatorname{dom}(L)\backslash\operatorname {Ker}(L)$, if $|D_{0+}^{\alpha-1}u(t)|+|D_{0+}^{\alpha-2}u(t)|>A$ for all $t\in [0,1]$, then
  $$Q^{\beta}_{1}N_{2}u(t)\neq0 \quad\mbox{or}\quad Q^{\beta}_{2}N_{2}u(t)\neq0, $$
  and if $|D_{0+}^{\beta-1}\upsilon(t)|+|D_{0+}^{\beta-2}\upsilon (t)|>A$ for all $t\in[0,1]$, then
  $$Q^{\alpha}_{1}N_{1}\upsilon(t)\neq0 \quad\mbox{or} \quad Q^{\alpha}_{2}N_{1}\upsilon (t)\neq0. $$
($H_{2}$)
There exist functions $a_{1},b_{1},d_{1},e_{1},r_{1}\in L^{1}[0,1]$ and a constant $\theta_{1}\in[0,1)$ such that, for all $(x,y,z)\in\mathbb{R}^{3}$ and a.e. $t\in [0,1]$, one of the following inequalities is satisfied:
  3.2 $$\begin{aligned} & \bigl\vert f(t,x,y,z) \bigr\vert \leq a_{1}(t) \vert x \vert+b_{1}(t) \vert y \vert+d_{1}(t) \vert z \vert+e_{1}(t) \vert z \vert^{\theta_{1}} +r_{1}(t), \end{aligned}$$
  3.3 $$\begin{aligned} & \bigl\vert f(t,x,y,z) \bigr\vert \leq a_{1}(t) \vert x \vert+b_{1}(t) \vert y \vert+d_{1}(t) \vert z \vert+e_{1}(t) \vert y \vert^{\theta_{1}} +r_{1}(t), \end{aligned}$$
  3.4 $$\begin{aligned} & \bigl\vert f(t,x,y,z) \bigr\vert \leq a_{1}(t) \vert x \vert+b_{1}(t) \vert y \vert+d_{1}(t) \vert z \vert+e_{1}(t) \vert x \vert^{\theta_{1}} +r_{1}(t). \end{aligned}$$
  There exist functions $a_{2},b_{2},d_{2},e_{2},r_{2}\in L^{1}[0,1]$ and a constant $\theta_{2}\in[0,1)$ such that, for all $(x,y,z)\in\mathbb{R}^{3}$ and a.e. $t\in [0,1]$, one of the following inequalities is satisfied:
  3.5 $$\begin{aligned} & \bigl\vert g(t,x,y,z) \bigr\vert \leq a_{2}(t) \vert x \vert+b_{2}(t) \vert y \vert+d_{2}(t) \vert z \vert+e_{2}(t) \vert z \vert^{\theta_{2}} +r_{2}(t), \end{aligned}$$
  3.6 $$\begin{aligned} & \bigl\vert g(t,x,y,z) \bigr\vert \leq a_{2}(t) \vert x \vert+b_{2}(t) \vert y \vert+d_{2}(t) \vert z \vert+e_{2}(t) \vert y \vert^{\theta_{2}} +r_{2}(t), \end{aligned}$$
  3.7 $$\begin{aligned} & \bigl\vert g(t,x,y,z) \bigr\vert \leq a_{2}(t) \vert x \vert+b_{2}(t) \vert y \vert+d_{2}(t) \vert z \vert+e_{2}(t) \vert x \vert^{\theta_{2}} +r_{2}(t). \end{aligned}$$
($H_{3}$)
There exists a constant $B>0$ such that, for every $c_{1},c_{2},c_{3},c_{4}\in\mathbb{R}$ satisfying $\sum_{i=1}^{4}c_{i}^{2}>B$, at least one of the following expressions holds:
  $$\begin{aligned} &R^{\alpha}_{1}N_{1} \bigl(c_{3}t^{\beta-1}+c_{4}t^{\beta-2} \bigr)\neq0,\qquad R^{\alpha}_{2}N_{1} \bigl(c_{3}t^{\beta-1}+c_{4}t^{\beta-2} \bigr)\neq0, \\ &R^{\beta}_{1}N_{2} \bigl(c_{1}t^{\alpha-1}+c_{2}t^{\alpha-2} \bigr)\neq0,\qquad R^{\beta}_{2}N_{2} \bigl(c_{1}t^{\alpha-1}+c_{2}t^{\alpha-2} \bigr)\neq0. \end{aligned}$$
  And for $c_{1},c_{2},c_{3},c_{4}\in\mathbb{R}$ satisfying $\sum_{i=1}^{4}c_{i}^{2}>B$,
  3.8 $$\begin{aligned} R\doteq{}& \bigl[c_{1}R^{\alpha}_{1}N_{1} \bigl(c_{3}t^{\beta-1}+c_{4}t^{\beta-2} \bigr)+c_{2}R^{\alpha}_{2}N_{1} \bigl(c_{3}t^{\beta-1}+c_{4}t^{\beta-2} \bigr) \bigr] \\ &{}\times \bigl[c_{3}R^{\beta}_{1}N_{2} \bigl(c_{1}t^{\alpha-1}+c_{2}t^{\alpha-2} \bigr)+c_{4}R^{\beta}_{2}N_{2} \bigl(c_{1}t^{\alpha-1}+c_{2}t^{\alpha-2} \bigr) \bigr] \leq0. \end{aligned}$$

### Remark 3.1

$R^{\alpha}_{i}N_{1}(at^{\beta-1}+bt^{\beta-2})$ and $R^{\beta}_{i}N_{2}(at^{\alpha-1}+bt^{\alpha-2})$ from $(H_{3})$ stand for the images of $\upsilon(t)=at^{\beta-1}+bt^{\beta-2},u(t)=at^{\alpha -1}+bt^{\alpha-2}$ under the maps $R^{\alpha}_{i}N_{1}$ and $R^{\beta}_{i}N_{2}$, respectively.

### Theorem 3.1

*If* $(H_{1})$*–*$(H_{3})$ *hold*, *then the coupled system of fractional multi*-*point boundary value problem* (1.2)*–*(1.3) *has at least one solution provided that*

3.9 $$\begin{aligned} &\max \bigl\{ \epsilon_{1} \bigl( \Vert a_{1} \Vert _{1}+ \Vert b_{1} \Vert _{1}+ \Vert d_{1} \Vert _{1} \bigr), \epsilon_{2} \bigl( \Vert a_{2} \Vert _{1}+ \Vert b_{2} \Vert _{1}+ \Vert d_{2} \Vert _{1} \bigr), \\ &\quad\rho_{1} \bigl( \Vert a_{1} \Vert _{1}+ \Vert b_{1} \Vert _{1}+ \Vert d_{1} \Vert _{1} \bigr)+\mu_{2} \bigl( \Vert a_{2} \Vert _{1}+ \Vert b_{2} \Vert _{1}+ \Vert d_{2} \Vert _{1} \bigr), \\ &\quad \rho_{2} \bigl( \Vert a_{1} \Vert _{1}+ \Vert b_{1} \Vert _{1}+ \Vert d_{1} \Vert _{1} \bigr)+\mu_{1} \bigl( \Vert a_{2} \Vert _{1}+ \Vert b_{2} \Vert _{1}+ \Vert d_{2} \Vert _{1} \bigr) \bigr\} < 1. \end{aligned}$$

### Proof

Our proof can be divided into four steps.

*Step* 1: Set

3.10 $$ \Omega_{1}= \bigl\{ (u,\upsilon)\in\operatorname {dom}(L)\backslash \operatorname{Ker}(L)|L(u,\upsilon)=\lambda N(u,\upsilon) \mbox{ for some } \lambda\in[0,1] \bigr\} . $$

Then, for $(u,\upsilon)\in\Omega_{1},L(u,\upsilon)=\lambda N(u,\upsilon)$, thus $\lambda\neq0,N(u,\upsilon)\in \operatorname{Im}(L)=\operatorname{Ker}(Q)$, hence $QN(u,\upsilon)=(Q^{\alpha}N_{1}\upsilon,Q^{\beta}N_{2}u)=(0,0)$ by the definition of *Q*. Thus we have $Q_{1}^{\alpha}N_{1}\upsilon(t)=Q^{\alpha}_{2}N_{1}\upsilon(t)=0$ and $Q_{1}^{\beta}N_{2}u(t)=Q^{\beta}_{2}N_{2}u(t)=0$ for all $t\in[0,1]$. It follows from $(H_{1})$ that there exist $t_{0},t_{1}\in[0,1]$ such that $|D_{0+}^{\alpha-1}u(t_{0})|+|D_{0+}^{\alpha-2}u(t_{0})|\leq A$ and $|D_{0+}^{\beta-1}\upsilon(t_{1})|+|D_{0+}^{\beta-2}\upsilon (t_{1})|\leq A$. Now

$$\begin{aligned} &D_{0+}^{\alpha-1}u(t)=D_{0+}^{\alpha-1}u(t_{0})+ \int_{t_{0}}^{t}D_{0+}^{\alpha}u(s) \, \mathrm{d}s, \\ &D_{0+}^{\alpha-2}u(t)=D_{0+}^{\alpha-2}u(t_{0})+ \int_{t_{0}}^{t}D_{0+}^{\alpha-1}u(s) \, \mathrm{d}s, \end{aligned}$$

so that

3.11 $$\begin{aligned} & \bigl\vert D_{0+}^{\alpha-1}u(0) \bigr\vert \leq \bigl\Vert D_{0+}^{\alpha-1}u(t) \bigr\Vert _{\infty}\leq \bigl\vert D_{0+}^{\alpha-1}u(t_{0}) \bigr\vert + \bigl\Vert D_{0+}^{\alpha}u \bigr\Vert _{1} \\ &\phantom{ \bigl\vert D_{0+}^{\alpha-1}u(0) \bigr\vert }\leq A+ \Vert Lu \Vert _{1}\leq A+ \Vert N_{1} \upsilon \Vert _{1}, \end{aligned}$$

3.12 $$\begin{aligned} & \bigl\vert D_{0+}^{\alpha-2}u(0) \bigr\vert \leq \bigl\Vert D_{0+}^{\alpha-2}u(t) \bigr\Vert _{\infty}\leq \bigl\vert D_{0+}^{\alpha-2}u(t_{0}) \bigr\vert + \bigl\Vert D_{0+}^{\alpha-1}u \bigr\Vert _{\infty} \\ &\phantom{ \bigl\vert D_{0+}^{\alpha-2}u(0) \bigr\vert } \leq \bigl\vert D_{0+}^{\alpha-2}u(t_{0}) \bigr\vert + \bigl\vert D_{0+}^{\alpha-1}u(t_{0}) \bigr\vert + \bigl\Vert D_{0+}^{\alpha}u \bigr\Vert _{1} \\ &\phantom{\phantom{ \bigl\vert D_{0+}^{\alpha-2}u(0) \bigr\vert }}\leq A+ \Vert L_{1}u \Vert _{1}\leq A+ \Vert N_{1}\upsilon \Vert _{1}. \end{aligned}$$

Similar to the above argument, we can also obtain

3.13 $$\begin{aligned} & \bigl\vert D_{0+}^{\beta-1}\upsilon(0) \bigr\vert \leq A+ \Vert N_{2}u \Vert _{1}, \end{aligned}$$

3.14 $$\begin{aligned} & \bigl\vert D_{0+}^{\beta-2}\upsilon(0) \bigr\vert \leq A+ \Vert N_{2}u \Vert _{1}. \end{aligned}$$

Now by (3.11)–(3.14) and (2.31), we have

3.15 $$\begin{aligned} \bigl\Vert P(u,\upsilon) \bigr\Vert _{Y}\leq{}& \max \biggl\{ \biggl(2+\frac{1}{\Gamma(\alpha)} \biggr) \bigl\vert D_{0+}^{\alpha-1}u(0) \bigr\vert + \biggl(1+\frac{1}{\Gamma(\alpha-1)} \biggr) \bigl\vert D_{0+}^{\alpha-2}u(0) \bigr\vert , \\ &{} \biggl(2+\frac{1}{\Gamma(\beta)} \biggr) \bigl\vert D_{0+}^{\beta-1} \upsilon(0) \bigr\vert + \biggl(1+\frac{1}{\Gamma(\beta-1)} \biggr) \bigl\vert D_{0+}^{\beta-2} \upsilon(0) \bigr\vert \biggr\} \\ \leq{}&\max \biggl\{ \biggl(3+\frac{1}{\Gamma(\alpha)}+\frac {1}{\Gamma(\alpha-1)} \biggr) \Vert N_{1}\upsilon \Vert _{1}+A \biggl(3+ \frac{1}{\Gamma(\alpha)}+ \frac{1}{\Gamma(\alpha-1)} \biggr), \\ &{} \biggl(3+\frac{1}{\Gamma(\beta)}+\frac{1}{\Gamma(\beta -1)} \biggr) \Vert N_{2}u \Vert _{1}+A \biggl(3+\frac{1}{\Gamma(\beta)}+ \frac{1}{\Gamma(\beta-1)} \biggr) \biggr\} . \end{aligned}$$

Note that $(I-P)(u,\upsilon)\in \operatorname{Im}(K_{P})=\operatorname{dom}(L)\cap \operatorname{Ker}(P)$ for $(u,\upsilon)\in\Omega_{1}$. Then

3.16 $$\begin{aligned} \bigl\Vert (I-P) (u,\upsilon) \bigr\Vert _{Y}&= \bigl\Vert K_{P}L(I-P) (u,\upsilon) \bigr\Vert _{Y} \\ &= \bigl\Vert K_{P} \bigl(L_{1}u,L^{2}\upsilon \bigr) \bigr\Vert _{Y} \\ &\leq \max \biggl\{ \biggl(2+\frac{1}{\Gamma(\alpha)} \biggr) \Vert L_{1}u \Vert _{1}, \biggl(2+\frac{1}{\Gamma(\beta)} \biggr) \Vert L_{2} \upsilon \Vert _{1} \biggr\} \\ &\leq \max \biggl\{ \biggl(2+\frac{1}{\Gamma(\alpha)} \biggr) \Vert N_{1} \upsilon \Vert _{1}, \biggl(2+\frac{1}{\Gamma(\beta)} \biggr) \Vert N_{2}u \Vert _{1} \biggr\} . \end{aligned}$$

Using (3.15) and (3.16), we obtain

3.17 $$\begin{aligned} \bigl\Vert (u,\upsilon) \bigr\Vert _{Y}={}& \bigl\Vert P(u,\upsilon)+(I-P) (u,\upsilon) \bigr\Vert _{Y}\leq \bigl\Vert P(u,\upsilon) \bigr\Vert _{Y}+ \bigl\Vert (I-P) (u, \upsilon) \bigr\Vert _{Y} \\ \leq{}&\max \biggl\{ \biggl(3+\frac{1}{\Gamma(\alpha)}+\frac {1}{\Gamma(\alpha-1)} \biggr) \Vert N_{1}\upsilon \Vert _{1}+A \biggl(3+ \frac{1}{\Gamma(\alpha)}+ \frac{1}{\Gamma(\alpha-1)} \biggr), \\ &{} \biggl(3+\frac{1}{\Gamma(\beta)}+\frac{1}{\Gamma(\beta -1)} \biggr) \Vert N_{2}u \Vert _{1}+A \biggl(3+\frac{1}{\Gamma(\beta)}+ \frac{1}{\Gamma(\beta-1)} \biggr) \biggr\} \\ &{} +\max \biggl\{ \biggl(2+\frac{1}{\Gamma(\alpha)} \biggr) \Vert N_{1} \upsilon \Vert _{1}, \biggl(2+\frac{1}{\Gamma(\beta)} \biggr) \Vert N_{2}u \Vert _{1} \biggr\} \\ ={}&\max \bigl\{ \epsilon_{1} \Vert N_{1}\upsilon \Vert _{1}+\rho_{1}A,\rho_{1} \Vert N_{1} \upsilon \Vert _{1}+\mu_{2} \Vert N_{2}u \Vert _{1}+\rho_{1}A, \\ &{} \epsilon_{2} \Vert N_{2}u \Vert _{1}+ \rho_{2}A,\rho_{2} \Vert N_{2}u \Vert _{1}+\mu_{1} \Vert N_{1}\upsilon \Vert _{1}+\rho_{2}A \bigr\} . \end{aligned}$$

Without loss of generality, we assume that (3.2) and (3.5) in $(H_{1})$ hold, then from (3.17), the proof can be divided into four cases.

*Case* 1. $\|(u,\upsilon)\|_{Y}\leq\epsilon_{1}\|N_{1}\upsilon\|_{1}+\rho_{1}A$. From (3.2), we have

3.18 $$\begin{aligned} \bigl\Vert (u,\upsilon) \bigr\Vert _{Y} \leq{}& \epsilon_{1} \bigl[ \Vert a_{1} \Vert _{1} \Vert \upsilon \Vert _{\infty}+ \Vert b_{1} \Vert _{1} \bigl\Vert D_{0+}^{\beta-2}\upsilon \bigr\Vert _{\infty}+ \Vert d_{1} \Vert _{1} \bigl\Vert D_{0+}^{\beta-1}\upsilon \bigr\Vert _{\infty} \\ &{}+ \Vert e_{1} \Vert _{1} \bigl\Vert D_{0+}^{\beta-1}\upsilon \bigr\Vert _{\infty}^{\theta_{1}}+ \Vert r_{1} \Vert _{1} \bigr]+\rho_{1}A. \end{aligned}$$

Thus, from $\|\upsilon\|_{\infty},\|D_{0+}^{\beta-2}\upsilon\|_{\infty}, \|D_{0+}^{\beta-1}\upsilon\|_{\infty}\leq\|(u,\upsilon)\|_{Y}$, and (3.17), we obtain

3.19 $$\begin{aligned} \Vert \upsilon \Vert _{\infty}\leq{}&\frac {1}{1-\epsilon_{1} \Vert a_{1} \Vert _{1}} \bigl[\epsilon_{1} \Vert b_{1} \Vert _{1} \bigl\Vert D_{0+}^{\beta-2}\upsilon \bigr\Vert _{\infty}+ \epsilon_{1} \Vert d_{1} \Vert _{1} \bigl\Vert D_{0+}^{\beta-1}\upsilon \bigr\Vert _{\infty} \\ &{} +\epsilon_{1} \Vert e_{1} \Vert _{1} \bigl\Vert D_{0+}^{\beta-1}\upsilon \bigr\Vert _{\infty}^{\theta_{1}}+ \epsilon_{1} \Vert r_{1} \Vert _{1}+ \rho_{1}A \bigr]. \end{aligned}$$

Again from (3.18), (3.19), one has

3.20 $$\begin{aligned} & \bigl\Vert D_{0+}^{\beta-2}\upsilon \bigr\Vert _{\infty}\leq\frac{1}{1-\epsilon_{1} \Vert a_{1} \Vert _{1}-\epsilon_{1} \Vert b_{1} \Vert _{1}} \bigl[ \epsilon_{1} \Vert d_{1} \Vert _{1} \bigl\Vert D_{0+}^{\beta-1} \upsilon \bigr\Vert _{\infty} \\ &\phantom{ \Vert D_{0+}^{\beta-2}\upsilon \Vert _{\infty}\leq}{}+\epsilon_{1} \Vert e_{1} \Vert _{1} \bigl\Vert D_{0+}^{\beta-1}\upsilon \bigr\Vert _{\infty}^{\theta_{1}}+\epsilon_{1} \Vert r_{1} \Vert _{1}+\rho_{1}A \bigr], \end{aligned}$$

3.21 $$\begin{aligned} & \bigl\Vert D_{0+}^{\beta-1}\upsilon \bigr\Vert _{\infty}\leq\frac{1}{1-\epsilon_{1} \Vert a_{1} \Vert _{1}-\epsilon _{1} \Vert b_{1} \Vert _{1}-\epsilon_{1} \Vert d_{1} \Vert _{1}} \\ &\phantom{ \Vert D_{0+}^{\beta-1}\upsilon \Vert _{\infty}\leq}{} \times \bigl[\epsilon_{1} \Vert e_{1} \Vert _{1} \bigl\Vert D_{0+}^{\beta-1}\upsilon \bigr\Vert _{\infty}^{\theta_{1}}+\epsilon_{1} \Vert r_{1} \Vert _{1}+\rho_{1}A \bigr]. \end{aligned}$$

Since $\theta_{1}\in[0,1)$, from the above last inequality, there exists $M_{1}>0$ such that $\|D_{0+}^{\beta-1}\upsilon\|_{\infty}\leq M_{1}$, thus from (3.20), there exists $M_{2}>0$ such that $\|D_{0+}^{\beta-2}\upsilon\|_{\infty}\leq M_{2}$. Again from (3.19), there exists $M_{3}>0$ such that $\|\upsilon\|_{\infty}\leq M_{3}$. Thus, from (3.18), there exists $M_{4}>0$ such that $\|(u,\upsilon)\|_{Y}\leq M_{4}$. Therefore $\Omega_{1}$ is bounded.

*Case* 2. $\|(u,\upsilon)\|_{Y}\leq\epsilon_{2}\|N_{2}u\|_{1}+\rho_{2}A$. The proof is similar to that of case 1. Here, we omit it.

*Case* 3. $\|(u,\upsilon)\|_{Y}\leq\rho_{1}\|N_{1}\upsilon\|_{1}+\mu_{2}\|N_{2}u\| _{1}+\rho_{1}A$. From (3.2) and (3.5), we have

3.22 $$\begin{aligned} \bigl\Vert (u,\upsilon) \bigr\Vert _{Y} \leq{}& \rho_{1} \bigl[ \Vert a_{1} \Vert _{1} \Vert \upsilon \Vert _{\infty}+ \Vert b_{1} \Vert _{1} \bigl\Vert D_{0+}^{\beta-2}\upsilon \bigr\Vert _{\infty}+ \Vert d_{1} \Vert _{1} \bigl\Vert D_{0+}^{\beta-1} \upsilon \bigr\Vert _{\infty} \\ &{} + \Vert e_{1} \Vert _{1} \bigl\Vert D_{0+}^{\beta-1}\upsilon \bigr\Vert _{\infty}^{\theta_{1}}+ \Vert r \Vert _{1} \bigr] \\ &{}+\mu_{2} \bigl[ \Vert a_{2} \Vert _{1} \Vert u \Vert _{\infty}+ \Vert b_{2} \Vert _{1} \bigl\Vert D_{0+}^{\alpha-2}u \bigr\Vert _{\infty}+ \Vert d_{2} \Vert _{1} \bigl\Vert D_{0+}^{\alpha-1}u \bigr\Vert _{\infty} \\ &{} +{} \Vert e_{2} \Vert _{1} \bigl\Vert D_{0+}^{\alpha-1}u \bigr\Vert _{\infty}^{\theta_{2}}+ \Vert r_{2} \Vert _{1} \bigr]+\rho_{1}A. \end{aligned}$$

Thus, from $\|u\|_{\infty},\|D_{0+}^{\alpha-2}u\|_{\infty},\|D_{0+}^{\alpha-1}u\| _{\infty},\|\upsilon\|_{\infty}, \|D_{0+}^{\beta-2}\upsilon\|_{\infty},\|D_{0+}^{\beta-1}\upsilon\| _{\infty}\leq\|(u,\upsilon)\|_{Y}$, and (3.22), we obtain

3.23 $$\begin{aligned} \Vert \upsilon \Vert _{\infty}\leq{}&\frac{1}{1-\rho _{1} \Vert a_{1} \Vert _{1}} \bigl[\rho_{1} \Vert b_{1} \Vert _{1} \bigl\Vert D_{0+}^{\beta-2}\upsilon \bigr\Vert _{\infty}+ \rho_{1} \Vert d_{1} \Vert _{1} \bigl\Vert D_{0+}^{\beta-1}\upsilon \bigr\Vert _{\infty} \\ &{} +\rho_{1} \Vert e_{1} \Vert _{1} \bigl\Vert D_{0+}^{\beta-1}\upsilon \bigr\Vert _{\infty}^{\theta_{1}}+ \rho_{1} \Vert r \Vert _{1} \\ &{}+\mu_{2} \Vert a_{2} \Vert _{1} \Vert u \Vert _{\infty}+\mu_{2} \Vert b_{2} \Vert _{1} \bigl\Vert D_{0+}^{\alpha-2}u \bigr\Vert _{\infty}+ \mu_{2} \Vert d_{2} \Vert _{1} \bigl\Vert D_{0+}^{\alpha-1}u \bigr\Vert _{\infty} \\ &{}+\mu_{2} \Vert e_{2} \Vert _{1} \bigl\Vert D_{0+}^{\alpha-1}u \bigr\Vert _{\infty}^{\theta_{2}}+ \mu_{2} \Vert r_{2} \Vert _{1}+ \rho_{1}A \bigr]. \end{aligned}$$

Again, from (3.22), (3.23), we have

3.24 $$\begin{aligned} &\Vert u \Vert _{\infty}\leq\frac{1}{1-\rho_{1} \Vert a_{1} \Vert _{1}-\mu_{2} \Vert a_{2} \Vert _{1}} \bigl[ \rho_{1} \Vert b_{1} \Vert _{1} \bigl\Vert D_{0+}^{\beta-2}\upsilon \bigr\Vert _{\infty}+ \rho_{1} \Vert d_{1} \Vert _{1} \bigl\Vert D_{0+}^{\beta-1}\upsilon \bigr\Vert _{\infty} \\ &\phantom{ \Vert u \Vert _{\infty}\leq}{} +\rho_{1} \Vert e_{1} \Vert _{1} \bigl\Vert D_{0+}^{\beta-1}\upsilon \bigr\Vert _{\infty}^{\theta_{1}}+ \rho_{1} \Vert r \Vert _{1} \\ &\phantom{ \Vert u \Vert _{\infty}\leq}{}+\mu_{2} \Vert b_{2} \Vert _{1} \bigl\Vert D_{0+}^{\alpha-2}u \bigr\Vert _{\infty}+ \mu_{2} \Vert d_{2} \Vert _{1} \bigl\Vert D_{0+}^{\alpha-1}u \bigr\Vert _{\infty} \\ &\phantom{ \Vert u \Vert _{\infty}\leq} {}+\mu_{2} \Vert e_{2} \Vert _{1} \bigl\Vert D_{0+}^{\alpha-1}u \bigr\Vert _{\infty}^{\theta_{2}}+ \mu_{2} \Vert r_{2} \Vert _{1}+ \rho_{1}A \bigr], \end{aligned}$$

3.25 $$\begin{aligned} & \bigl\Vert D_{0+}^{\beta-2}\upsilon \bigr\Vert _{\infty}\leq \frac{1}{1-\rho_{1} \Vert a_{1} \Vert _{1}-\rho_{1} \Vert b_{1} \Vert _{1}-\mu_{2} \Vert a_{2} \Vert _{1}} \bigl[ \rho_{1} \Vert d_{1} \Vert _{1} \bigl\Vert D_{0+}^{\beta-1} \upsilon \bigr\Vert _{\infty} \\ &\phantom{ \Vert D_{0+}^{\beta-2}\upsilon \Vert _{\infty}\leq}{} +\rho_{1} \Vert e_{1} \Vert _{1} \bigl\Vert D_{0+}^{\beta-1}\upsilon \bigr\Vert _{\infty}^{\theta_{1}}+ \rho_{1} \Vert r \Vert _{1} \\ &\phantom{ \Vert D_{0+}^{\beta-2}\upsilon \Vert _{\infty}\leq}{}+\mu_{2} \Vert b_{2} \Vert _{1} \bigl\Vert D_{0+}^{\alpha-2}u \bigr\Vert _{\infty}+ \mu_{2} \Vert d_{2} \Vert _{1} \bigl\Vert D_{0+}^{\alpha-1}u \bigr\Vert _{\infty} \\ &\phantom{ \Vert D_{0+}^{\beta-2}\upsilon \Vert _{\infty}\leq}{} +\mu_{2} \Vert e_{2} \Vert _{1} \bigl\Vert D_{0+}^{\alpha-1}u \bigr\Vert _{\infty}^{\theta_{2}}+ \mu_{2} \Vert r_{2} \Vert _{1}+ \rho_{1}A \bigr], \end{aligned}$$

3.26 $$\begin{aligned} & \bigl\Vert D_{0+}^{\alpha-2}u \bigr\Vert _{\infty}\leq \frac{1}{1-\rho_{1} \Vert a_{1} \Vert _{1}-\rho_{1} \Vert b_{1} \Vert _{1}-\mu_{2} \Vert a_{2} \Vert _{1}-\mu_{2} \Vert b_{2} \Vert _{1}} \bigl[ \rho_{1} \Vert d_{1} \Vert _{1} \bigl\Vert D_{0+}^{\beta-1} \upsilon \bigr\Vert _{\infty} \\ &\phantom{ \Vert D_{0+}^{\alpha-2}u \Vert _{\infty}\leq }{} +\rho_{1} \Vert e_{1} \Vert _{1} \bigl\Vert D_{0+}^{\beta-1}\upsilon \bigr\Vert _{\infty}^{\theta_{1}}+ \rho_{1} \Vert r \Vert _{1} +\mu_{2} \Vert d_{2} \Vert _{1} \bigl\Vert D_{0+}^{\alpha-1}u \bigr\Vert _{\infty} \\ &\phantom{ \Vert D_{0+}^{\alpha-2}u \Vert _{\infty}\leq }{} +\mu_{2} \Vert e_{2} \Vert _{1} \bigl\Vert D_{0+}^{\alpha-1}u \bigr\Vert _{\infty}^{\theta_{2}}+ \mu_{2} \Vert r_{2} \Vert _{1}+ \rho_{1}A \bigr] \end{aligned}$$

and

3.27 $$\begin{aligned} & \bigl\Vert D_{0+}^{\alpha-1}u \bigr\Vert _{\infty}\leq \frac{1}{1-\rho_{1}( \Vert a_{1} \Vert _{1}+ \Vert b_{1} \Vert _{1}+ \Vert d_{1} \Vert )-\mu_{2}( \Vert a_{2} \Vert _{1}+ \Vert b_{2} \Vert _{1}+ \Vert d_{2} \Vert _{1})} \\ &\phantom{ \Vert D_{0+}^{\alpha-1}u \Vert _{\infty}\leq }{}\times \bigl[\rho_{1} \Vert e_{1} \Vert _{1} \bigl\Vert D_{0+}^{\beta-1}\upsilon \bigr\Vert _{\infty}^{\theta_{1}} +\mu_{2} \Vert e_{2} \Vert _{1} \bigl\Vert D_{0+}^{\alpha-1}u \bigr\Vert _{\infty}^{\theta_{2}} \\ &\phantom{ \Vert D_{0+}^{\alpha-1}u \Vert _{\infty}\leq }{} +\mu_{2} \Vert r_{2} \Vert _{1}+ \rho_{1} \bigl(A+ \Vert r_{1} \Vert _{1} \bigr) \bigr], \end{aligned}$$

3.28 $$\begin{aligned} & \bigl\Vert D_{0+}^{\beta-1}\upsilon \bigr\Vert _{\infty}\leq \frac{1}{1-\rho_{1}( \Vert a_{1} \Vert _{1}+ \Vert b_{1} \Vert _{1}+ \Vert d_{1} \Vert )-\mu_{2}( \Vert a_{2} \Vert _{1}+ \Vert b_{2} \Vert _{1}+ \Vert d_{2} \Vert _{1})} \\ &\phantom{ \Vert D_{0+}^{\beta-1}\upsilon \Vert _{\infty}\leq }{}\times \bigl[\rho_{1} \Vert e_{1} \Vert _{1} \bigl\Vert D_{0+}^{\beta-1}\upsilon \bigr\Vert _{\infty}^{\theta_{1}} +\mu_{2} \Vert e_{2} \Vert _{1} \bigl\Vert D_{0+}^{\alpha-1}u \bigr\Vert _{\infty}^{\theta_{2}} \\ &\phantom{ \Vert D_{0+}^{\beta-1}\upsilon \Vert _{\infty}\leq }{}+\mu_{2} \Vert r_{2} \Vert _{1}+ \rho_{1} \bigl(A+ \Vert r_{1} \Vert _{1} \bigr) \bigr]. \end{aligned}$$

If $\rho_{1}\|e_{1}\|_{1}\|D_{0+}^{\beta-1}\upsilon\|_{\infty}^{\theta_{1}} \geq\mu_{2}\|e_{2}\|_{1}\|D_{0+}^{\alpha-1}u\|_{\infty}^{\theta_{2}}$, then from (3.28) we have

3.29 $$\begin{aligned} \bigl\Vert D_{0+}^{\beta-1}\upsilon \bigr\Vert _{\infty}\leq{}&\frac{1}{1-\rho_{1}( \Vert a_{1} \Vert _{1}+ \Vert b_{1} \Vert _{1}+ \Vert d_{1} \Vert )-\mu_{2}( \Vert a_{2} \Vert _{1}+ \Vert b_{2} \Vert _{1}+ \Vert d_{2} \Vert _{1})} \\ &{}\times \bigl[2\rho_{1} \Vert e_{1} \Vert _{1} \bigl\Vert D_{0+}^{\beta-1}\upsilon \bigr\Vert _{\infty}^{\theta_{1}}+\mu_{2} \Vert r_{2} \Vert _{1}+\rho_{1} \bigl(A+ \Vert r_{1} \Vert _{1} \bigr) \bigr]. \end{aligned}$$

Since $\theta_{1}\in[0,1)$, from the above last inequality, there exists $M_{1}>0$ such that $\|D_{0+}^{\beta-1}\upsilon\|_{\infty}\leq M_{1}$, thus from (3.22)–(3.28), there exists $M_{2}>0$ such that $\|u\|_{\infty}$, $\|\upsilon\|_{\infty}$, $\|D_{0+}^{\alpha-2}u\| _{\infty}$, $\|D_{0+}^{\beta-2}\upsilon\|_{\infty}$, and $\|D_{0+}^{\alpha-1}u\|_{\infty}$ are all less than $M_{2}$, hence $\|(u,\upsilon)\|_{Y}\leq3(M_{1}+M_{2})$. Therefore $\Omega_{1}$ is bounded.

If $\rho_{1}\|e_{1}\|_{1}\|D_{0+}^{\beta-1}\upsilon\|_{\infty}^{\theta_{1}} \leq\mu_{2}\|e_{2}\|_{1}\|D_{0+}^{\alpha-1}u\|_{\infty}^{\theta_{2}}$, then from (3.27), similar to the above argument, we can also prove that $\Omega_{1}$ is bounded.

*Case* 4. $\|(u,\upsilon)\|_{Y}\leq\rho_{2}\|N_{2}u\|_{1}+\mu_{1}\|N_{1}\upsilon\| _{1}+\rho_{2}A$. The proof is similar to that of case 3. Here, we omit it.

From the above argument, we have proved that $\Omega_{1}$ is bounded.

*Step* 2: Let

$$\Omega_{2}=\bigl\{ (u,\upsilon)\in\operatorname{Ker}(L)|N(u,\upsilon) \in \operatorname{Im}(L)\bigr\} . $$

For $(u,\upsilon)\in\Omega_{2},(u,\upsilon)\in\operatorname {Ker}(L)=\{(u,\upsilon)\in \operatorname{dom}(L)|u=c_{11}t^{\alpha-1}+c_{12}t^{\alpha -2},\upsilon=c_{21}t^{\beta-1}+c_{22}t^{\beta-2}, c_{ij}\in\mathbb {R},i,j=1,2,t\in [0,1]\}$, and $QN(c_{11}t^{\alpha-1}+c_{12}t^{\alpha -2},c_{21}t^{\beta-1}+c_{22}t^{\beta-2})=(0,0)$, thus

$$\begin{aligned} &R^{\alpha}_{1}N_{1}\bigl(c_{21}t^{\beta-1}+c_{22}t^{\beta-2} \bigr)=R^{\alpha}_{2}N_{1}\bigl(c_{21}t^{\beta-1}+c_{22}t^{\beta-2} \bigr)=0, \\ &R^{\beta}_{1}N_{2}\bigl(c_{11}t^{\alpha-1}+c_{12}t^{\alpha-2} \bigr)=R^{\alpha}_{2}N_{2}\bigl(c_{11}t^{\alpha-1}+c_{12}t^{\alpha-2} \bigr)=0. \end{aligned}$$

By $(H_{3})$, $c_{11}^{2}+c_{12}^{2}+c_{21}^{2}+c_{22}^{2}\leq B$, that is, $\Omega_{2}$ is bounded.

*Step* 3: We define the isomorphism $J:\operatorname{Im}(Q)\rightarrow \operatorname{Ker}(L)$ by

$$\begin{aligned} &J \bigl(c_{11}t^{\alpha-1}+c_{12}t^{\alpha-2},c_{21}t^{\beta -1}+c_{22}t^{\beta-2} \bigr) \\ &\quad = \bigl(c_{11}t^{\alpha-1}+c_{12}t^{\alpha-2},c_{21}t^{\beta -1}+c_{22}t^{\beta-2} \bigr), \quad c_{ij}\in\mathbb{R},i,j=1,2. \end{aligned}$$

Let

$$\Omega_{3}=\bigl\{ (u,\upsilon)\in\operatorname{Ker}(L)|-\lambda J^{-1} (u,\upsilon)+(1-\lambda)QN(u,\upsilon)=(0,0),\lambda\in[0,1] \bigr\} . $$

For every $(c_{11}t^{\alpha-1}+c_{12}t^{\alpha-2},c_{21}t^{\beta -1}+c_{22}t^{\beta-2})\in\Omega_{3}$ with $c_{11}^{2}+c_{12}^{2}+c_{21}^{2}+c_{22}^{2}>0$,

$$\begin{aligned} &\lambda \bigl(c_{11}t^{\alpha-1}+c_{12}t^{\alpha-2},c_{21}t^{\beta -1}+c_{22}t^{\beta-2} \bigr) \\ &\quad =(1-\lambda) \bigl(Q^{\alpha}N_{1}\upsilon,Q^{\beta}N_{2} u \bigr) \\ &\quad=(1-\lambda) \bigl( \bigl(R^{\alpha}_{1}N_{1} \bigl(c_{21}t^{\beta-1}+c_{22}t^{\beta-2} \bigr) \bigr)t^{\alpha-1} + \bigl(R^{\alpha}_{2}N_{1} \bigl(c_{21}t^{\beta-1}+c_{22}t^{\beta-2} \bigr) \bigr)t^{\alpha-2}, \\ &\qquad \bigl(R^{\beta}_{1}N_{2} \bigl(c_{11}t^{\alpha-1}+c_{12}t^{\alpha-2} \bigr) \bigr)t^{\beta-1} + \bigl(R^{\beta}_{2}N_{2} \bigl(c_{11}t^{\alpha-1}+c_{12}t^{\alpha-2} \bigr) \bigr)t^{\beta-2} \bigr). \end{aligned}$$

If $\lambda=1$, then $c_{ij}=0,i,j=1,2$. If $\lambda=0$, then by Step 2, $c_{11}^{2}+c_{12}^{2}+c_{21}^{2}+c_{22}^{2}< B$. If $0<\lambda<1$ and $c_{11}^{2}+c_{12}^{2}+c_{21}^{2}+c_{22}^{2}>B$, then by $(H_{3})$,

$$\begin{aligned} &\lambda^{2} \bigl(c_{11}^{2}+c_{12}^{2}+c_{21}^{2}+c_{22}^{2} \bigr) \\ &\quad=(1-\lambda)^{2} \bigl[c_{11} \bigl(R^{\alpha}_{1}N_{1} \bigl(c_{21}t^{\beta-1}+c_{22}t^{\beta-2} \bigr) \bigr) +c_{12} \bigl(R^{\alpha}_{2}N_{1} \bigl(c_{21}t^{\beta-1}+c_{22}t^{\beta-2} \bigr) \bigr) \bigr] \\ &\qquad{}\times \bigl[c_{21} \bigl(R^{\beta}_{1}N_{2} \bigl(c_{11}t^{\alpha-1}+c_{12}t^{\alpha-2} \bigr) \bigr) +c_{22} \bigl(R^{\beta}_{2}N_{2} \bigl(c_{11}t^{\alpha-1}+c_{12}t^{\alpha-2} \bigr) \bigr) \bigr]\leq0, \end{aligned}$$

which, in either case, is a contradiction, that is, $\Omega_{3}$ is bounded.

*Step* 4: Now we prove that the conditions of Theorem 2.1 are all satisfied. Set Ω to be a bounded open set of *Y* such that $\bigcup_{i=1}^{3}\overline{\Omega}_{i}\subset\Omega$. By Lemma 2.3, the operator $K_{P}(I-Q)N:\overline{\Omega}\rightarrow Y$ is compact, thus *N* is *L*-compact on Ω̅. Then, by the above argument, we have

(i) $L(u,\upsilon)) \neq\lambda N(u,\upsilon)$ for every $((u,\upsilon),\lambda) \in [(\operatorname{dom}(L)\backslash\operatorname{Ker}(L))\cap\partial \Omega]\times(0,1)$;
(ii) $N(u,\upsilon)\notin \operatorname{Im}(L)$ for every $(u,\upsilon)\in \operatorname{Ker}(L)\cap \partial\Omega$.

Let $H((u,\upsilon),\lambda)= \lambda I (u,\upsilon)+(1-\lambda)JQN(u,\upsilon)$, where *I* is the identical operator. According to the above argument, we know

$$H\bigl((u,\upsilon),\lambda\bigr)\neq0, \quad\mbox{for all } (u,\upsilon)\in \operatorname{Ker}(L)\cap \partial\Omega, $$

thus, by the homotopy property of degree

$$\begin{aligned} &\operatorname{deg} \bigl(JQN|_{\operatorname{Ker}(L)},\Omega\cap \operatorname{Ker}(L),(0,0) \bigr) \\ &\quad =\operatorname{deg} \bigl(H( \cdot,0),\Omega\cap\operatorname{Ker}(L),(0,0) \bigr) \\ &\quad =\operatorname{deg} \bigl(H(\cdot,1),\Omega\cap\operatorname {Ker}(L),(0,0) \bigr) =\operatorname{deg} \bigl(I,\Omega\cap \operatorname{Ker}(L),(0,0) \bigr)\neq0. \end{aligned}$$

Thus (iii) of Theorem 2.1 is satisfied. Then, by Theorem 2.1, $L(u,\upsilon)=N(u,\upsilon)$ has at least one solution in $\operatorname{dom}(L)\cap\overline{\Omega}$, so that the coupled system (1.2)–(1.3) has at least one solution in *Y*. The proof is finished. □

## Conclusions

The linear operator $L=0$ with boundary conditions at resonance with the kernel of four dimensions was considered and an existence result for a coupled system of nonlinear fractional differential equations with multi-point boundary conditions at resonance was obtained by using the coincidence degree theory.
